# Examining the role of paraoxonase 2 in the dopaminergic system of the mouse brain

**DOI:** 10.1186/s12868-022-00738-4

**Published:** 2022-09-02

**Authors:** Jacqueline M. Garrick, Khoi Dao, Lucio G. Costa, Judit Marsillach, Clement E. Furlong

**Affiliations:** 1 Department of Environmental and Occupational Health Sciences, University of Washington, Seattle, WA USA; 2 Department of Medicine and Surgery, University of Parma, Parma, Italy; 3 Departments of Medicine (Div. Medical Genetics) and of Genome Sciences, University of Washington, Seattle, USA

**Keywords:** Paraoxonase 2 (PON2), Transcript, Protein, Dopamine, Agonist, Antagonist

## Abstract

**Background:**

Paraoxonase 2 (PON2) is an intracellular antioxidant enzyme located at the inner mitochondrial membrane. Previous studies have found PON2 to be an important antioxidant in a variety of cellular systems, such as the cardiovascular and renal system. Recent work has also suggested that PON2 plays an important role in the central nervous system (CNS), as decreased PON2 expression in the CNS leads to higher oxidative stress and subsequent cell toxicity. However, the precise role of PON2 in the CNS is still largely unknown, and what role it may play in specific regions of the brain remains unexamined. Dopamine metabolism generates considerable oxidative stress and antioxidant function is critical to the survival of dopaminergic neurons, providing a potential mechanism for PON2 in the dopaminergic system.

**Methods:**

In this study, we investigated the role of PON2 in the dopaminergic system of the mouse brain by comparing transcript and protein expression of dopaminergic-related genes in wildtype (WT) and PON2 deficient (PON2-def) mouse striatum, and exposing WT cultured primary neurons to dopamine receptor agonists.

**Results:**

We found alterations in multiple key dopaminergic genes at the transcript level, however many of these changes were not observed at the protein level. In cultured neurons, PON2 mRNA and protein were increased upon exposure to quinpirole, a dopamine receptor 2/3 (DRD2/3) agonist, but not fenoldopam, a dopamine receptor 1/5 (DRD1/5) agonist, suggesting a receptor-specific role in dopamine signaling.

**Conclusions:**

Our findings suggest PON2 deficiency significantly impacts the dopaminergic system at the transcript level and may play a role in mitigating oxidative stress in this system further downstream through dopamine receptor signaling.

**Supplementary Information:**

The online version contains supplementary material available at 10.1186/s12868-022-00738-4.

## Background

Paraoxonase 2 (PON2) is a member of the paraoxonase family consisting of three genes: PON1, 2 and 3. All three genes align in tandem on mouse chromosome 6 and the long arm of human chromosome 7q21-22 [1]. Although the family was named for the esterase activity of PON1 against paraoxon, the active metabolite of the organophosphate insecticide parathion, PON2 possesses minimal esterase activity and does not hydrolyze paraoxon. However, all three PONs possess lactonase activity, exhibiting overlapping but distinct substrates for lactone hydrolysis [2]. While PON1 is a circulating enzyme associated with high-density lipoproteins (HDL) in plasma and PON3 both circulates and expresses intracellularly, PON2 does not circulate and is an intracellular, membrane-bound enzyme located at the inner mitochondrial membrane [3–5]. Here, PON2 associates with co-enzyme Q_10_ and is thought to play a role maintaining redox balance during oxidative phosphorylation [5]. In neurons and astrocytes, PON2 has also been found at the cell membrane [6], but the function of PON2 at this location, and whether it differs from that of the mitochondrial membrane, is unknown. It is ubiquitously expressed and observed in all tissues examined, with the highest expression found in the lung, liver, intestines, heart, brain and kidneys respectively [6]. PON2 expression has been reported as sexually dimorphic, with females displaying significantly higher levels of PON2 than males [7, 8]. However, this expression pattern has not been consistent, with some studies reporting no difference [9].

Previous studies have demonstrated PON2 is a critical antioxidant enzyme, as the loss of PON2 is associated with multiple morbidities in-vivo, such as atherosclerosis [5, 10], heart failure [11], impaired insulin signaling [12, 13], and obesity [14]. Epidemiological studies have also linked PON2 SNPs with Alzheimer’s disease [15], suggesting an important role in the brain. Although the mechanism by which PON2 acts as an antioxidant has yet to be fully understood, prevailing evidence supports PON2 acts by inhibiting semiquinone-mediated superoxide formation [16]. Inhibition of radical formation is of particular importance in the dopaminergic system of the central nervous system (CNS) where dopamine (DA) metabolism generates free radicals and reactive quinones through DA oxidation [17]. This continuous oxidative burden from DA metabolism renders dopaminergic neurons more vulnerable to additional oxidative stressors and cellular death [18]. Indeed, a leading hypothesis on the etiology of Parkinson’s disease (PD) suggests uncontrolled oxidative stress cascades leading to dopaminergic neuron death likely play a critical role in disease pathology [19]. This hypothesis is supported by significant in-vivo evidence of higher oxidative stress markers in the brains of human PD patients [20] and genetic mouse models [21]. Neurotoxicant animal models mimic PD via exposure to 6-hydroxydopamine (6-OHDA), 1-methyl-4-phenyl-1,2,3,6-tetrahydropyridine (MPTP), or agropesticides which cause mitochondrial dysfunction and/or oxidative stress to destroy dopaminergic neurons [19], highlighting the importance of both redox balance and mitochondrial function for survival and proper function of dopaminergic neurons.

PON2 has been shown to be differentially expressed in the brain, with mice having significantly higher PON2 in dopaminergic regions such as the striatum and substantia nigra [6]. In-vitro PON2 has been shown to interact with DJ-1 (PARK7) and the protective antioxidant effects of DJ-1 are mediated partly through PON2 expression [22]. DJ-1 is implicated in PD etiology and loss-of-function mutations of DJ-1 account for 1% of all familial PD cases; whether PON2 plays a role in PD etiology is currently unknown and no additional studies related to PON2 and PD exist in the literature. In the kidney, studies have shown PON2 plays an important antioxidant role in the renal dopaminergic system, a critical system for maintaining homeostatic blood pressure [23]. Activation of dopamine receptors 2 and 5 (DRD2 and DRD5) upregulates PON2 expression which in turn inhibits renal NADPH oxidases, pro-oxidant enzymes that produce ROS [24, 25]. Maintenance of redox balance by antioxidants such as PON2 is important as unhampered ROS in the renal system can have detrimental effects to both electrolyte balance and systemic blood pressure.

Considering the expression pattern and antioxidant functionality of PON2, as well as evidence from other organ systems, it is feasible that PON2 may play an important role in the dopaminergic system of the CNS. Minimal studies have addressed the role of PON2 in the CNS, despite in-vivo and epidemiological evidence suggesting its importance. To address whether PON2 plays a role in this system, we examined wildtype (WT) and PON2 deficient (PON2-def) striatum from mice and compared the transcript and protein levels of key dopaminergic-related targets, to determine if PON2 deficiency leads to expression changes, specifically within the nigrostriatal dopaminergic system. The striatum was chosen as the tissue of interest as it is the primary active site of dopaminergic signaling for the nigrostriatal pathway, where dopamine synthesis and release occurs in the synaptic terminals of innervating neurons from the substantia nigra pars compacta (SNc), and the receiving dopamine receptors lie on the striatal interneurons. Additionally, we conducted in-vitro experiments with cultured primary neurons to determine if PON2 expression was modulated upon exposure to two selective dopamine receptor (DR) agonists, fenoldopam (DRD1/5) and quinpirole (DRD2/3).

## Materials and methods

### Materials

Anti-DRD1, DRD2, DRD5, DAT, HO-1, HO-2, NOX2, TH and VMAT2 antibodies were purchased from Abcam (Cambridge, MA, USA). Anti-PON2 antibody was purchased from Genscript (Piscataway, NJ, USA). Anti β-actin antibody, papain, poly-d-lysine, quinpirole hydrochloride, fenoldopam mesylate and L-741,626 were purchased from MilliporeSigma (Burlington, MA, USA). Anti-rabbit IgG HRP-linked antibody and Cell Lysis Buffer 10 × were purchased from HRP Goat Anti-Mouse IgG was purchased from BD Biosciences (San Jose, CA, USA). XCell II Blot Module, XCell SureLock Electrophoresis Cell, NuPAGE MOPS SDS Running Buffer 20x, NuPAGE LDS Sample Buffer 4x, NuPAGE Antioxidant, NuPAGE Sample Reducing Agent 10x, NuPAGE 10% Bis–Tris Protein Gels, Neurobasal-A medium, B-27 supplement with and without antioxidant, fungizone, HBSS medium (No Ca^2+^) and 10 mM HEPES were purchased from Life Technologies (Carlsbad, CA, USA). Immobilon-P Transfer Membrane was purchased from Millipore Corporation (Billerica, MA, USA). Restore Western Blot Stripping Buffer, PageRuler Prestained Protein Ladder, SuperSignal West Pico Chemiluminescent Substrate and Costar 6-well TC plates were purchased from Thermo Fisher Scientific (Waltham, MA, USA). RNeasy Mini Kit was purchased from Qiagen (Hilden, Germany). iScript cDNA Synthesis Kit, iTaq Universal SYBR Green Supermix and CFX384 Real-Time Detection System were purchases from Bio-Rad Laboratories (Hercules, CA, USA). GraphPad Prism software version 9.1.2 was purchased from GraphPad Software Inc. (San Diego, CA, USA).

### Animals, tissue and cell culture

#### Mice

Wild-type (WT) and PON2 deficient (PON2-def) mice [10] on a C57BL/6 J background were used for this study. *N* = 5 mice per sex per genotype were utilized, with male and female mice combined for analysis if no sex difference was observed. Mice were euthanized at postnatal day (PND) 60 using carbon dioxide (CO_2_) followed by decapitation. Striatal tissue was dissected and flash frozen in liquid nitrogen. Tissue was pulverized with a pre-chilled mortar and pestle into a fine powder, stored at − 80 °C and aliquoted into appropriate lysis buffers for protein and RNA extraction as needed, utilizing sonication for homogenization when indicated. Mice were housed in a specific pathogen-free facility on a 14-h light/ 10-h dark cycle with ad libitum access to food and water. All procedures were conducted in accordance with the National Institute of Health Guide for the Use and Care of Laboratory Animals and were approved by the University of Washington Institutional Animal Care and Use Committee (IACUC) under protocol #2077-14. Animal numbers were kept to a minimum and all efforts were made to reduce animal suffering.

#### Primary cell culture

Cerebellar granule neurons (CGNs) were prepared from PND 7 mice, as previously described [26]. A total of 3–5 pups were pooled per cell preparation, with male and female pups prepared separately. In this study, 4 cell preparations (2 female, 2 male) were used. Neurons were grown for 10–12 days before treatments. Briefly, cerebellar tissue was collected in chilled HBSS medium (No Ca^2+^) and 10 mM HEPES. Tissues were digested for 30 min in HBSS containing papain (1 mg/mL) and DNAse I (40 μg/mL) and centrifuged at 300 × *g* for 7 min at room temperature. The supernatant (containing papain) was removed, and the pellet was gently triturated in serum-free Neurobasal-A Medium supplemented with B27 (Life Technologies, Carlsbad, CA). Cells were centrifuged at 200 × *g* at 4 °C for 10 min and the cell pellet was gently resuspended in medium. Neurons were then counted, seeded on poly-D-lysine coated 6-well plates at a density of 5 × 10^4^/ cm^2^, and cultured in serum-free Neurobasal-A medium supplemented with B27. After 4 days, media was completely exchanged for serum-free Neurobasal-A Medium with B27 minus antioxidants (−AO). Concentrations for the agonist/antagonist experiments were determined from a set of preliminary experiments (data not shown), with a starting concentration of 1 μM chosen from existing literature [24, 25]. 1 μM quinpirole was not sufficient to increase PON2 expression in primary neurons, while 3 μM and 20 μM quinpirole were. 1, 3 and 20 μM fenoldopam did not affect PON2 expression. 3 μM quinpirole was chosen for the remaining experiments as it was the lowest concentration which modulated PON2 expression in our preliminary examination, and no difference in expression was found between 3 μM and 20 μM quinpirole. Quinpirole in low doses (≤ 100 nM) is demonstrated to selectively target autoreceptors in-vivo when injected into mice [27, 28]. All of our investigated concentrations are considered high by existing literature standards and are anticipated to reduce autoreceptor binding and preferentially target postsynaptic receptors. For the DRD1 and DRD2 agonist experiments, neurons were treated for 24 h with fenoldopam mesylate (3 μM) or quinpirole hydrochloride (3 μM). For the DRD2 antagonist experiment, neurons were pre-treated for 1 h with L-741,626 (3 μM) prior to a 24-h co-incubation with L-741,626 (3 μM) and quinpirole (3 μM) together.

### Immunoblotting

Immunoblots were carried out as previously described [29]. Briefly, 5 mg of pulverized striatum was homogenized in 1 × Cell Lysis Buffer (Cell Signaling Technology, Danvers, MA) and 15 µg of protein was mixed with SDS running buffer and sample reducing agent and subjected to sodium dodecyl sulfate–polyacrylamide gel electrophoresis (SDS-PAGE). Following electrophoresis, proteins were transferred to polyvinylidene difluoride membranes, and the membrane blocked for 1–3 h with 5% nonfat milk. Membranes were then probed with the following diluted primary antibodies: PON2 1:2000, DRD1 1:10,000, DRD2 1:2500, DRD5 1:2500, TH 1:3000, VMAT2 1:1500, DAT 1:1000, HO-1 1:5000, HO-2 1:3000 and NOX2 1:12,000. Following primary antibody incubation, membranes were washed with Tris-buffered saline with 0.1% Tween-20 (pH = 7.5) and incubated with horseradish peroxidase-conjugated anti-rabbit secondary antibody at the following dilutions: PON2 1:5000, DRD1 1:5000, DRD2 1:5000, DRD5 1:5000, TH 1:3000, VMAT2 1:3000, DAT 1:5000, HO-1 1:4000, HO-2 1:3000 and NOX2 1:4000. Membranes were stripped with Restore™ Western Blot Stripping Buffer (Thermo Fisher Scientific, Waltham, MA) and re-probed for β-actin using a dilution of 1:2500 for the β-actin primary antibody and 1:2500 for the horseradish peroxidase-conjugated anti-mouse secondary antibody. Intensity of bands was measured by densitometry using ImageJ software (NIH), with the band intensity normalized to β-actin expression.

### RT-PCR

Total RNA was extracted from striatal tissue using the RNeasy Mini Kit (Qiagen, Hilden, Germany) according to the manufacturer’s established protocol. Target mRNA levels were measured by RT-PCR, see Table 1 for sequences of primer pairs. cDNA was generated from 1 μg total RNA using the iScript cDNA Synthesis Kit (Bio-Rad Laboratories, Hercules, CA) according to the manufacture’s established protocol. The cDNA samples were diluted to a concentration of 6.25 ng/μL with nuclease-free water and subsequently used for quantitative polymerase chain reaction (qPCR) using iTaq Universal SYBR Green Supermix in a CFX384 Real-Time Detection System (Bio-Rad Laboratories, Hercules, CA). 6 µL of diluted cDNA were included in a PCR reaction mastermix containing 15 μL Universal SYBR Green Supermix (2x), 1.5 µL each forward and reverse primers (10 μM stock), and 6 µL nuclease-free water (30 µL final volume). The reaction mixture was then aliquoted in triplicate, 8 µL per reaction per sample. The thermal cycling conditions were as follows: A single denaturing step at 95 °C for 30 s, 40 cycles of 95 °C for 15 s, 60 °C for 30 s and 72 °C for 30 s. dCq values (referenced as “relative mRNA”) for each target were calculated by subtracting Cq values of housekeeping gene GAPDH. The stability of GAPDH was assessed using the NormFinder algorithm in Excel [30] and found to be stably expressed between WT and PON2-def tissue, in both males and females (Additional file 1).

**Table 1:** RT-PCR primer pair sequences

Target	Forward Primer (5’–3’)	Reverse Primer (5’–3’)
PON2	GCACGCTGGTGGACAATTTATC	TGTCACTGATGGCTTCTCGGAT
DRD1	TCTTTGTCATCTCTTTAGCTGTGTC	TTCGGAGTCATCTTCCTCTCA
DRD2	ATCTCTTGCCCACTGCTCTTTGGA	ATAGACCAGCAGGGTGACGATGAA
DRD5	ACCAAGACACGGTCTTCCAC	CCTCCTCCTCACAGTCAAGC
TH	CAGCTGGAGGATGTGTCTCA	GGCATGACGGATGTACTGTG
VMAT2	TCATCGCTGCAGGCTCCATCT	AGCTGCCACTTTCGGGAACAC
DAT	GGGTGGCCTGGTTCTACG	CAGCATAGCCGCCAGTACAG
HO-1	GCCGAGAATGCTGAGTTCATG	TGGTACAAGGAAGCCATCACC
HO-2	TACTTCACATACTCAGCCCT	ATGGGCCACCAGCAGCTCTG
NOX2	CAGGAACCTCACTTTCCATAAGATG	AACGTTGAAGAGATGTGCAATTGT
GAPDH	TGACCTCAACTACATGGTCTACA	CTTCCCATTCTCGGCCTTG

### Statistical analysis

Statistical analyses were conducted using GraphPad Prism software (version 9.1.2, GraphPad Software Inc., San Diego, CA, USA). Data are expressed as the mean ± SEM and all graphed results represent biological replicates. For immunoblotting and qPCR experiments, tissue from at least 5 animals per group was included. For primary cell culture experiments, 4 independent experiments were analyzed, with each experimental cell population derived from a different animal cohort. One-way ANOVA followed by the Bonferroni correction for multiple comparisons was utilized for statistical analysis of multiple groups, while Student’s t-test was utilized for comparing two groups as noted. Prior to ANOVA analysis, data normality was assessed using the Shapiro–Wilk test.

## Results

### Verification of PON2 deficiency

#### PON2 expression

**Fig. 1:**
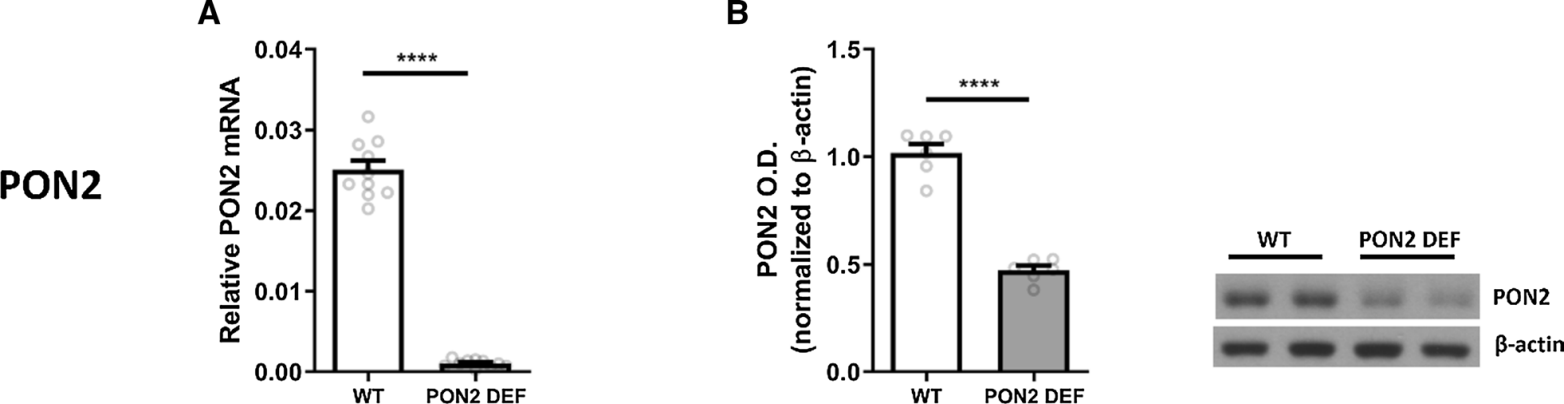
mRNA and Protein Expression of Paraoxonase 2 (PON2) in PON2 deficient mouse striatum. **A** Quantification of PON2 mRNA normalized to GAPDH, mean (± SEM), n = 10 per group, **** p < 0.0001. **B** Quantification of PON2 protein normalized to β-actin, mean (± SEM), n = 5 per group, **** p < 0.0001

To verify if the mice used for this study were PON2 deficient, we measured PON2 transcript and protein expression in WT and PON2-def mice. The PON2-def mice had nearly 30 times lower transcript expression of PON2 (Fig. 1A), and 2–3 times lower protein expression (Fig. 1B).

### Comparing dopaminergic targets in PON2 deficient and wildtype mice

#### Dopamine receptors

**Fig. 2:**
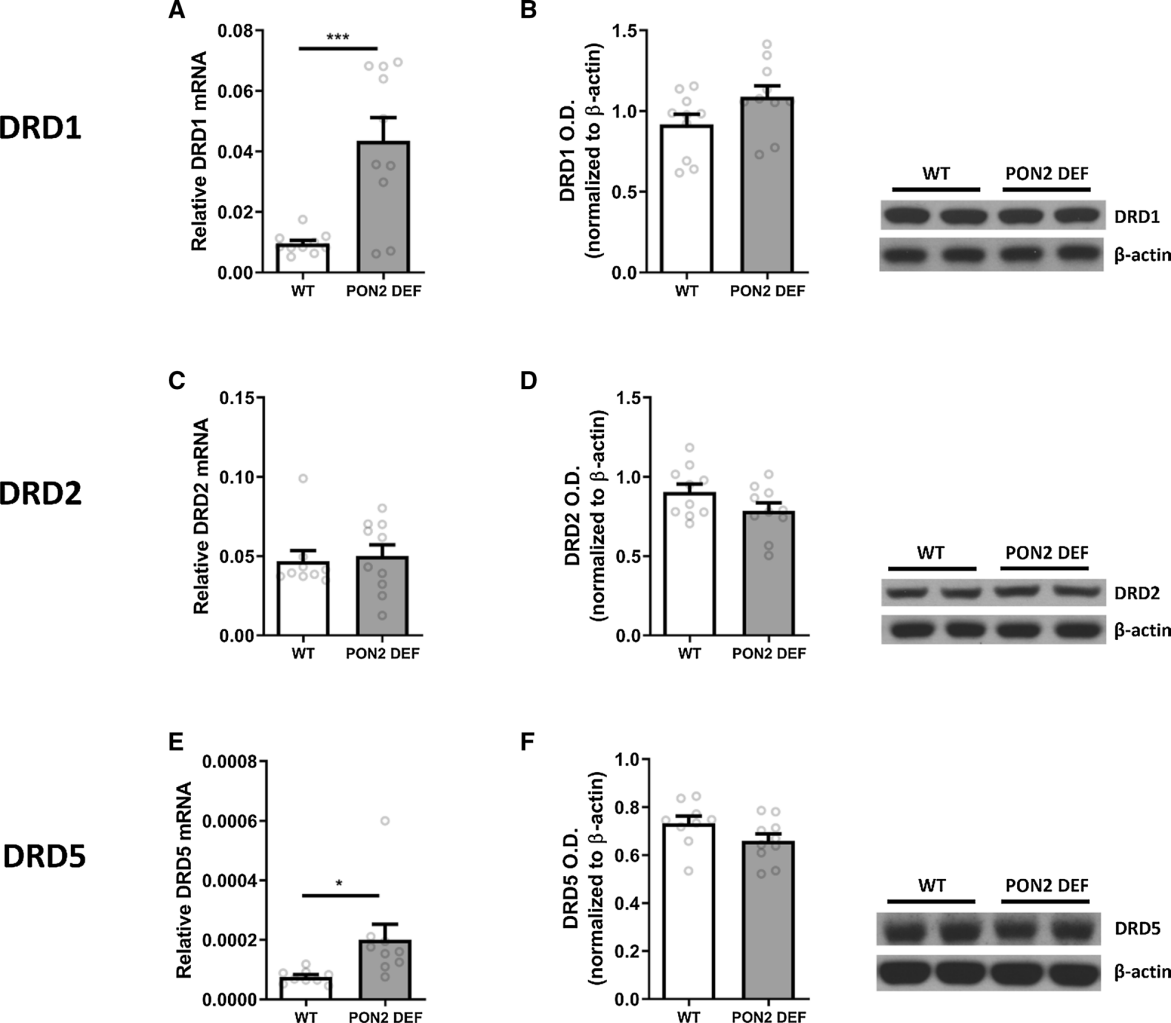
mRNA and Protein Expression of Dopamine Receptors in PON2 deficient mouse striatum. **A** Quantification of dopamine receptor 1 (DRD1) mRNA normalized to GAPDH, mean (± SEM), n = 9–10 per group, *** p < 0.001. **B** Quantification of dopamine receptor D1 (DRD1) protein normalized to β-actin, mean (± SEM), n = 8–10 per group. **C** Quantification of dopamine receptor D2 (DRD2) mRNA normalized to GAPDH, mean (± SEM), n = 9–10 per group. **D** Quantification of DRD2 protein normalized to β-actin, mean (± SEM), n = 9–10 per group. **E** Quantification of dopamine receptor D5 (DRD5) mRNA normalized to GAPDH, mean (± SEM), n = 9–10 per group, * p < 0.05. **F** Quantification of DRD5 protein normalized to β-actin, mean (± SEM), n = 9–10 per group

In the striatum, PON2-def mice had significantly higher levels of DRD1 (Fig. 2A) and DRD5 (Fig. 2E) transcript compared to WT mice, while DRD2 (Fig. 2C) levels were identical. To further investigate changes at the protein level, these targets were also measured using Western blot analysis. However, no differences were seen when comparing protein levels of any dopamine receptor D1, D2 and D5 respectively (Fig. 2B, D, F).

#### Dopamine metabolism and transport

**Fig. 3:**
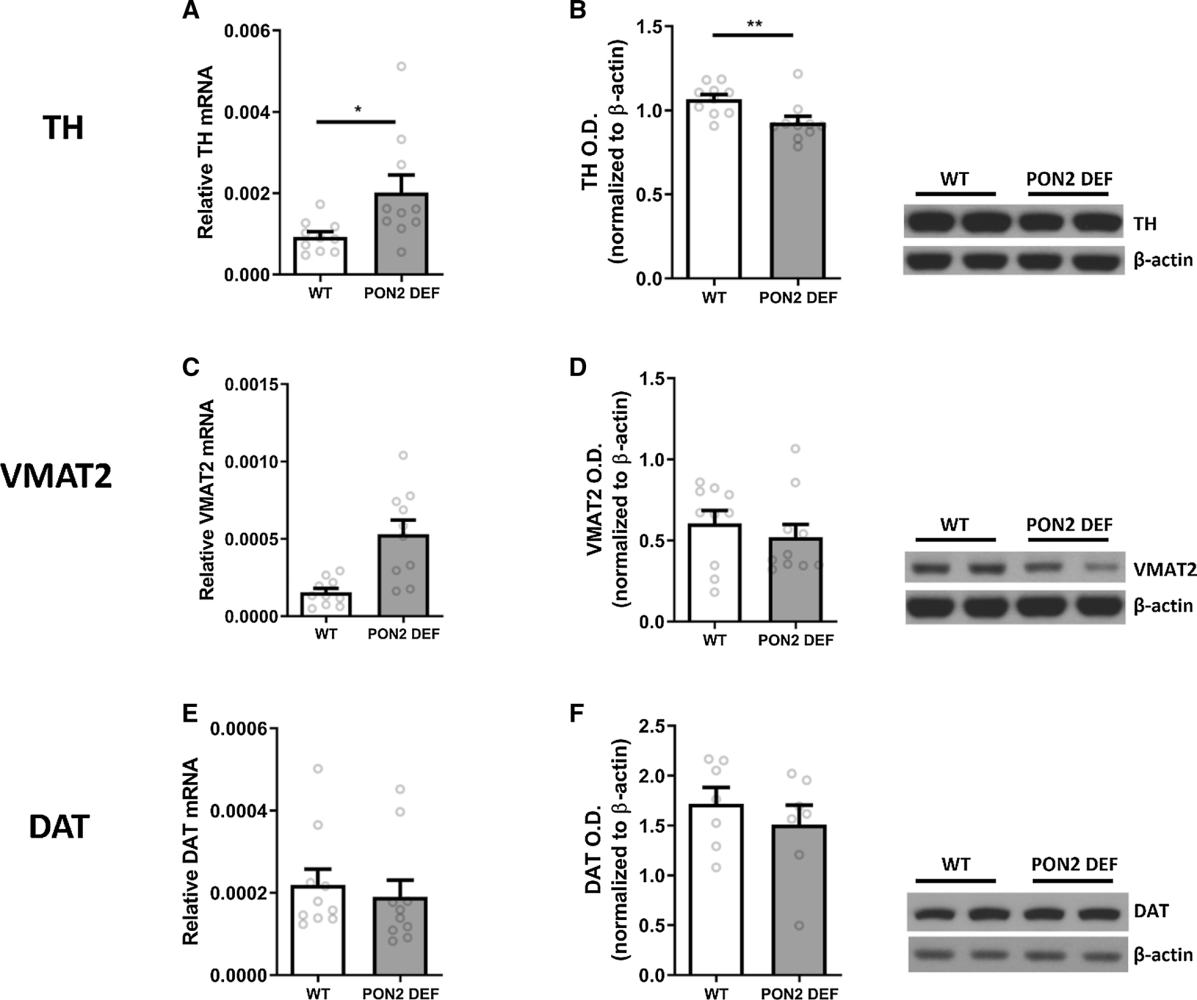
mRNA and protein expression of dopamine metabolism genes in PON2 deficient mouse striatum. **A** Quantification of tyrosine hydroxylase (TH) mRNA normalized to GAPDH, mean (± SEM), n = 8–10 per group, * p < 0.05. **B** Quantification of TH protein normalized to β-actin, mean (± SEM), n = 8–10 per group. **p < 0.01 **C** Quantification of vesicular monoamine transporter 2 (VMAT2) mRNA normalized to GAPDH, mean (± SEM), n = 9–10 per group, *** p < 0.001 **D** Quantification of VMAT2 protein normalized to β-actin, mean (± SEM), n = 9–10 per group. **E** Quantification of dopamine transporter (DAT) mRNA normalized to GAPDH, mean (± SEM), n = 10 per group. **F** Quantification of DAT protein normalized to β-actin, mean (± SEM), n = 7–10 per group

Various genes involved in DA metabolism and transport were measured at the transcript and protein level using RT-PCR and Western blot analysis. Transcript for tyrosine hydroxylase (TH), the rate limiting enzyme in DA metabolism, was measured and found to be significantly higher in PON2-def striatum compared to WT (Fig. 3A), with an opposite effect observed in the measured protein levels, where PON2-def striatum had decreased TH protein compared to WT (Fig. 3B). Vesicular monoamine transporter 2 (VMAT2), the principle solute carrier which shuttles DA from the cytosol into synaptic vesicles for release at the presynaptic membrane, was found to have significantly higher transcript levels in PON2-def striatum compared to WT (Fig. 3C), although there were no significant differences in the protein levels (Fig. 3D). Dopamine transporter (DAT), the synaptic transmembrane transporter which recovers DA from the synaptic cleft back into the cytosol, was unchanged at both the transcript (Fig. 3E) and protein level (Fig. 3F).

#### Antioxidant enzymes

**Fig. 4:**
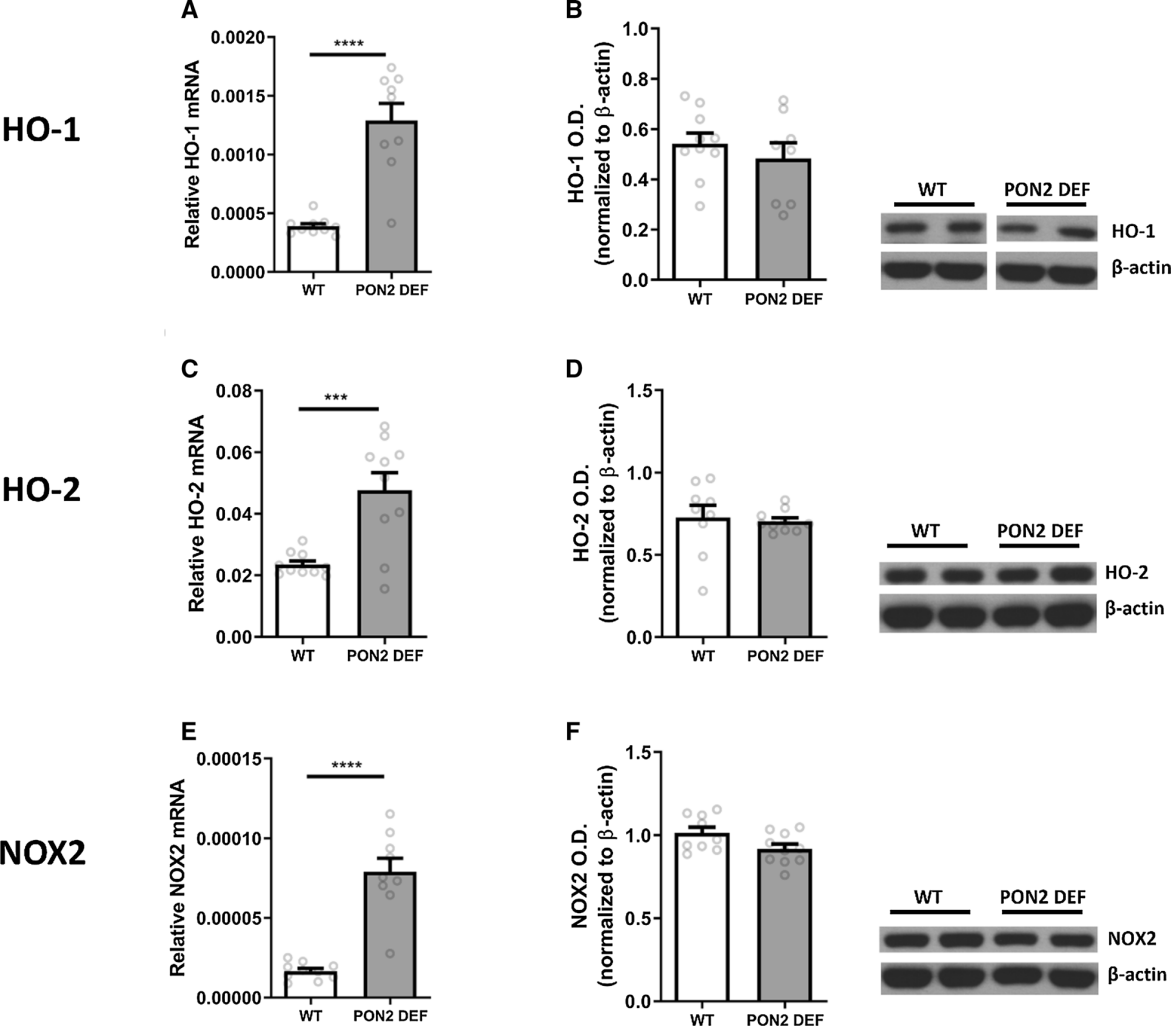
mRNA and Protein Expression of Antioxidant Genes in PON2 deficient mouse striatum. **A** Quantification of heme oxygenase 1 (HO-1) mRNA normalized to GAPDH, mean (± SEM), n = 9–10 per group, **** p < 0.0001. **B** Quantification of HO-1 protein normalized to β-actin, mean (± SEM), n = 8–10 per group. **C** Quantification of heme oxygenase 2 (HO-2) mRNA normalized to GAPDH, mean (± SEM), n = 9—10 per group, *** p < 0.001. **D** Quantification of HO-2 normalized to β-actin, mean (± SEM), n = 9–10 per group. **E** Quantification of NADPH oxidase 2 (NOX2) mRNA normalized to GAPDH, mean (± SEM), n = 8–10 per group, **** p < 0.0001. **F** Quantification of NOX2 protein normalized to β-actin, mean (± SEM), n = 8–9 per group

Oxidative balance is of particular importance in dopaminergic neurons, and we sought to determine if PON2 deficiency affects the expression of specific antioxidant enzymes in the striatum. Transcript levels of heme-oxygenase 1 (HO-1) and 2 (HO-2) were significantly higher in PON2-def striatum compared to WT (Fig. 4A, C), while no differences were observed at the protein level (Fig. 4B, D). Similarly, NADPH oxidase 2 (NOX2) transcript levels were significantly increased in PON2-def mice (Fig. 4E), while the protein levels of NOX2 were unchanged (Fig. 4F).

### PON2 expression in CGNs after dopamine receptor agonist exposure

#### Dopamine receptor D1 and D5 signaling

**Fig. 5:**
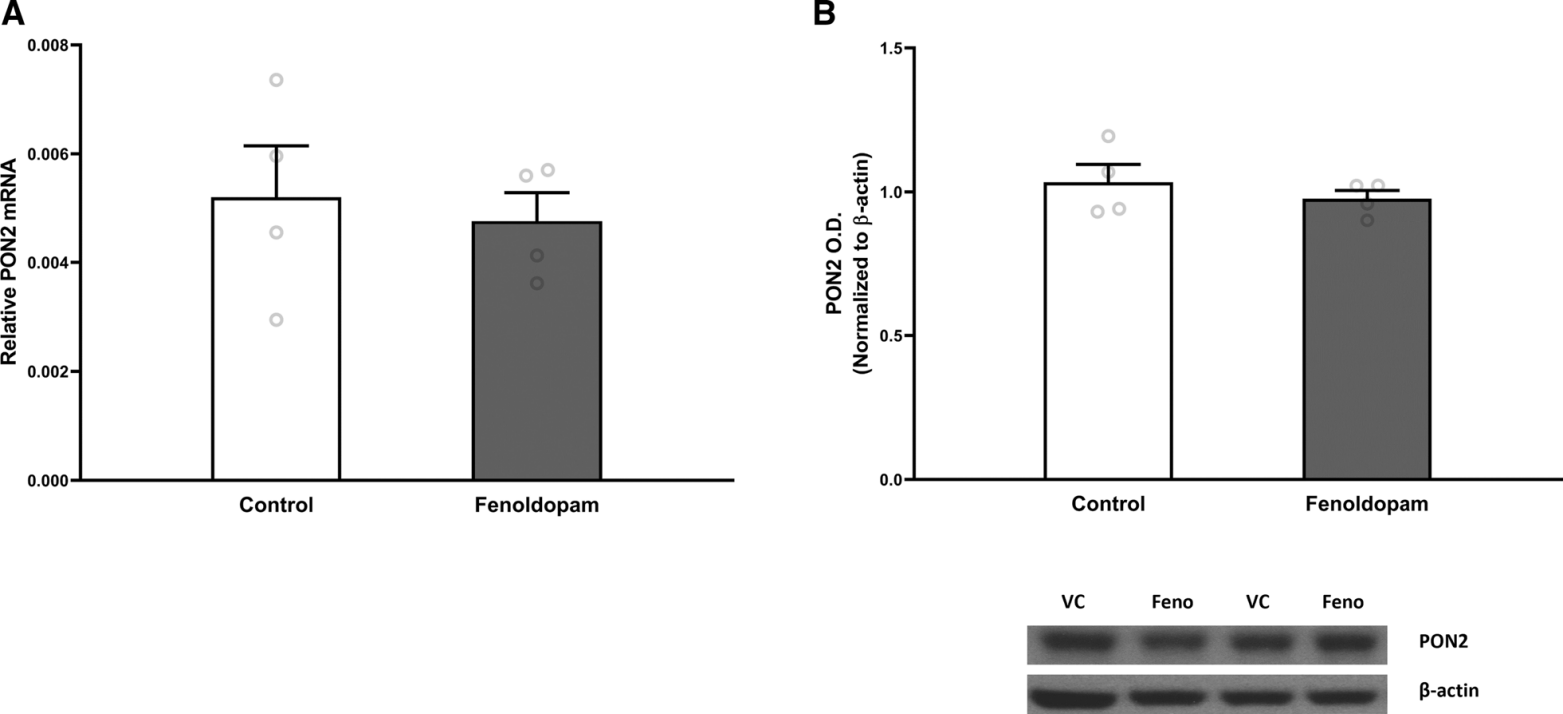
mRNA and Protein Expression of PON2 after Exposure to Dopamine Receptor 1/5 Agonist Fenoldopam. **A** Quantification of paraoxonase 2 (PON2) mRNA in cerebellar granule neurons after 24-h exposure to 3 μM fenoldopam, normalized to GAPDH, mean (± SEM), n = 4 per group **B** Quantification of paraoxonase 2 (PON2) protein in cerebellar granule neurons after 24-h exposure to 3 μM fenoldopam, normalized to β-actin, mean (± SEM), n = 4 per group

To investigate whether PON2 may be modulated by DA signaling, we exposed neurons to a DRD1/5 agonist, fenoldopam, and measured PON2 transcript and protein. A 24-h incubation with 3 μM fenoldopam did not alter PON2 expression compared to untreated cells at the transcript (Fig. 5A) or protein level (Fig. 5B). Higher concentrations of up to 20 μM also did not affect PON2 expression (data not shown).

### Dopamine receptor D2 signaling

**Fig. 6:**
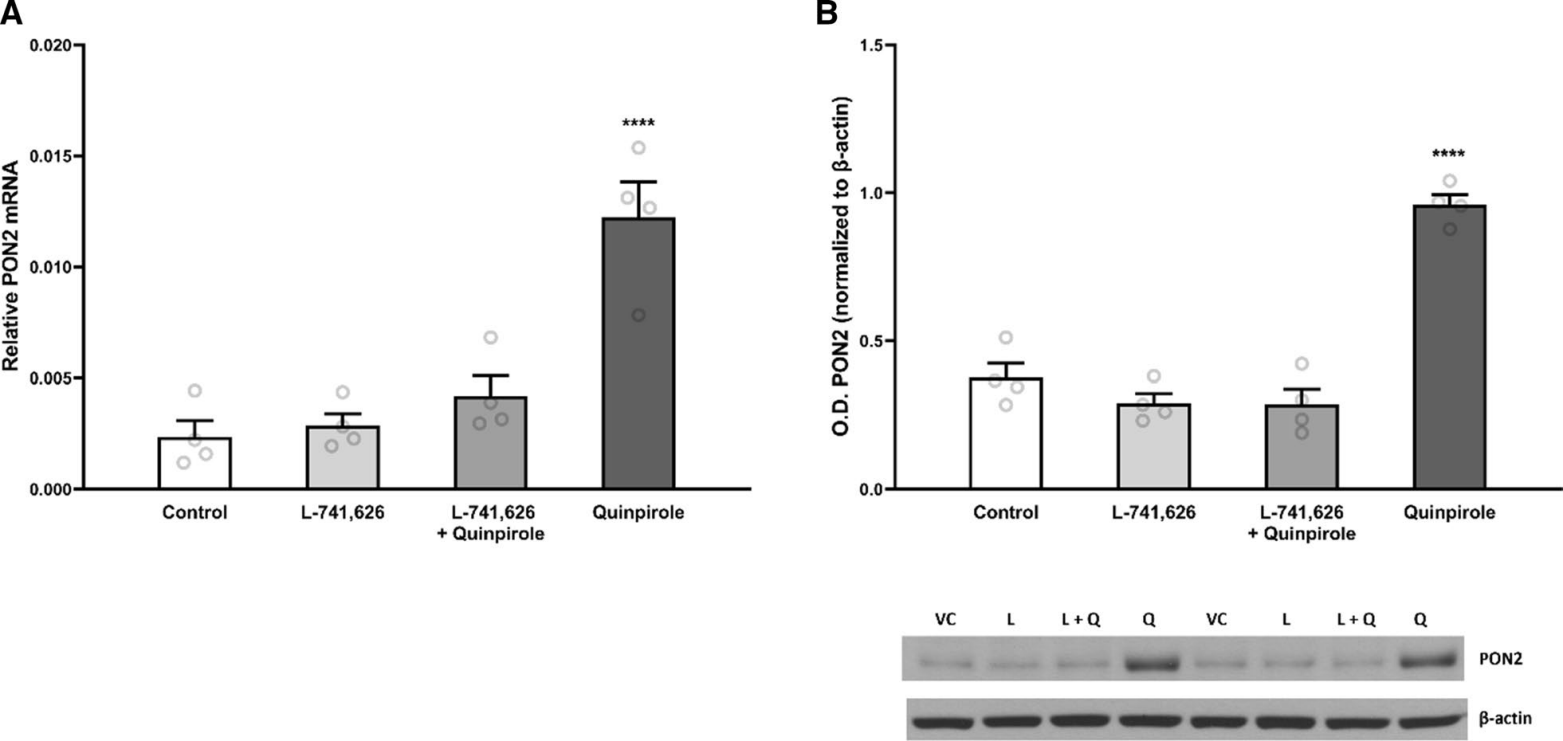
mRNA and Protein Expression of PON2 after Exposure to Dopamine Receptor 2/3 Agonist Quinpirole. **A** Quantification of paraoxonase 2 (PON2) mRNA in cerebellar granule neurons after 24-h exposure to 3 μM quinpirole or 3 μM dopamine receptor D2 antagonist L-741,626 alone or combined with 3 μM quinpirole, normalized to GAPDH, mean (± SEM), n = 4 per group **B** Quantification of paraoxonase 2 (PON2) protein in cerebellar granule neurons after 24-h exposure to 3 μM quinpirole or 3 μM dopamine receptor D2 antagonist L-741,626 alone or combined with 3 μM quinpirole, normalized to β-actin, mean (± SEM), n = 4 per group

We also evaluated whether PON2 could be modulated by DRD2 signaling by exposing primary neurons to the DRD2/3 agonist quinpirole. A 24-h incubation with 3 μM quinpirole significantly increased the expression of PON2 at both the transcript and protein level (Fig. 6A and B). A 1-h pre-treatment and 24 h co-exposure with a competitive DRD2 antagonist, L-741,626, was able to abolish the increased PON2 expression (Fig. 6A, B). These findings support that the observed expression increase of PON2 was due to activation of DRD2, and not that of DRD3 or non-selective binding to other targets.

## Discussion

While widely expressed in the brain, PON2 levels are the highest in dopaminergic regions such as the striatum, suggesting a function in these areas [6]. Feasibly, PON2 expression may be higher in these regions due to the increased oxidative stress burden stemming from DA metabolism, a process which generates ROS [17]. Using a PON2 deficient mouse model, we have shown that PON2 deficiency in the striatum significantly impacts multiple dopaminergic-related genes at the transcript level, upregulating DRD1, DRD5, TH and VMAT2. Transcript levels of antioxidant enzymes NOX2, HO-1 and HO-2 were also significantly increased in PON2-def mice, suggesting these animals may have higher burdens of oxidative stress in the striatum.

Although expressing significantly less PON2 transcript and protein compared to WT mice, the PON2-def mouse is not a full knockout model, with these animals producing a small amount of PON2. It is currently unknown what amount of PON2 loss is necessary before dysfunction occurs in a given system, and it is possible that this deficient model does not reach that threshold for overt impacts to the dopaminergic system. Moreover, it is unknown how stable this deficiency is over time. PON2-def mice may experience periods of oscillating deficiency over their lifetime, and our single-time point analysis in this study would be unable to capture such dynamic changes. To eliminate many of these issues, utilization of a full knockout model would be ideal for investigation of PON2 going forward, with incorporation of the PON2-def model for later analysis. Indeed, the deficient model does have translational investigation benefits, as PON2 polymorphisms identified in the general population are believed to reduce the enzymatic activity of PON2, but do not abolish its expression [31]. In the absence of a humanized knock-in model, the deficient model may be the best functional approximation of these polymorphisms to study the translational aspects of PON2.

Notably, no sex differences were observed in our study, and male and female data was subsequently combined for analysis. PON2 expression has been reported in the literature to be higher in females in both mice [7] and monkeys [8]. However, our present study did not identify differential expression of PON2 between WT females and males, and sex was not a modulating factor when comparing dopaminergic gene expression between WT and PON2-def mice. These results are consistent with a recent study from our group comparing brain region RNA expression between WT and PON2-def mice [9], where a sex difference in PON2 expression was also not observed. Estrogen is thought to be the driving factor behind sex-based PON2 expression differences, as removing the ovaries of female mice leads to a reduction in PON2 levels [7]. Potentially, the estrus cycle is an important regulator of PON2, and sex-based expression differences may be linked to the estrus cycle and fluctuations in circulating estrogen levels of female animals. Estrus cycle status of females was not accounted for in our study, but would be worthwhile to note for future studies. There is also evidence that PON2 expression may be under the control of circadian regulators [9], and the time of tissue collection could be an important factor for PON2 expression measurements.

Despite significant increases in transcript with PON2 deficiency, most targets were unchanged at the protein level in the striatum. In the case of TH, the transcript and protein effects observed were opposite and PON2-def mice may have a reduction in DA metabolism because of lower TH protein expression, although analysis of DA metabolites would be necessary to confirm this. In addition to metabolites, measurement of the DA catabolic pathway would be of interest for this tissue but was not fully addressed in our study. We have found no changes in monoamine oxidase B (MAOB) expression with PON2 deficiency (Additional file 2), but we have not investigated the expression of monoamine oxidase A (MAOA), the primary catabolizer of DA in rodents [32, 33]. Future studies probing the role of PON2 in this system would benefit from the inclusion of catabolic pathways to understand impacts to the full lifecycle of DA.

The reported discrepancies in transcript and protein could be the result of multiple factors and are not uncommon, as correlation between transcript and protein is known to be poor, with Pearson correlation coefficients reported as low as R = 0.36 [34]. Recent work looking at global transcript and protein expression in the human brain also supports this, identifying a Pearson correlation coefficient ranging from R = 0.32–0.5 [35]. An important factor for this poor correlation identified by Carlyle et al. is the vast neuronal projection network throughout the brain, where the cell bodies of neurons reside in one region and the synaptic terminals in another. This is of relevance for our study as well, as many of the targets we analyzed for DA metabolism are synaptic proteins. These synaptic terminals in the striatum originate from neurons in other regions of the brain, such as the substantia nigra pars compacta (SNc) in the case of dopaminergic input. Thus, our usage of striatal tissue includes the axons and synaptic terminals of SNc neurons but excludes their cell bodies. Our study did not look at the SNc, limiting our view of RNA expression within SNc neurons to transcripts which are locally translated at the synapse. Local transcript translation at the synapse is now recognized as an important function, particularly for neurons which project long distances and for which shuttling of proteins from the cell body down the axon may not be ideal for rapid signal transduction [36, 37]. TH has been experimentally shown to be locally translated [38], and other DA-related transcripts may follow suit for rapid signal transduction within this system. However, it is unknown when a transcript is preferentially translated locally, or when the protein is shuttled from the cell body. Since our study only analyzed striatal tissue, we are unable to comment on the expression changes that may be occurring in the SNc cell bodies of projected neurons. Expression data from the SNc would provide valuable insight into regional changes and identify potential discrepancies between translation occurring in the cell body versus the synaptic terminals. While RNA differences may be detected in striatal tissue, translation and shuttling of proteins may be occurring in the cell body from the SNc which negate any protein changes upon analysis of striatal tissue. As well, striatal tissue includes striatal interneurons which express TH but are not dopaminergic [39], which may influence our expression results while not reflecting the dopaminergic system. Although the size of the interneuron population is low, around 10% of striatal cells [40], our methodology does not allow us to separate expression data from projection neurons versus interneurons. Sub-analysis of these populations would be of interest to determine if PON2 plays a differential role in projection neurons and interneurons.

An additional explanation for the observed transcript-protein discrepancy may be a temporal element to changes in the dopaminergic system that our study was unable to capture. Increased transcript may be present for quick translation and rapid increase in protein levels under physiological conditions that were not present at the time our tissue was collected. While transcript is expected to predict steady-state protein levels, defining ‘steady-state’ presents a challenge as cells are constantly adapting to their environment and can be in numerous states of change in a given period, such as during proliferation, differentiation or apoptosis [41]. Our study utilized whole striatal tissue comprised of multiple neural cell types, ranging from non-proliferating neurons to highly dynamic glial cells. Astrocytes are reported to have significantly higher expression of PON2 compared to neurons [6], and there may be underlying expression differences between these cell populations. Further investigation of specific cell types would be of interest to determine if there are differences in neural and glial populations, as well as additional time points after oxidative challenge to determine if these increases in transcript are primed for an adaptive response. Additionally, these discrepancies may point to a dysregulation in transcription mediation in PON2-def mice, where transcription is increased but translational controls maintain constant protein levels. This could be the result of increased activity of specific transcription factors to elicit transcription upregulation, while micro RNA (miRNA) activity and *trans-*acting proteins prevent increases of protein [42]. Our lab has recently identified that PON2 deficiency alters the expression of multiple targets related to transcription in the mouse brain, which could affect the expression of the targets analyzed in this study [9]. Excess protein may also be targeted for degradation through the ubiquitin–proteasome pathway to maintain proteostasis if previous translational controls are insufficient [43]. While increased transcript levels may not pose a risk when these controls are in place, loss of translational and chaperone control is noted in aged animals [44]. If these higher transcript levels persist for the lifespan of the PON2 deficient animal, effects on the dopaminergic system may be more apparent in old age as these control systems break down and protein levels become aberrant. Increased expression of dopamine receptors may lead to signaling abnormalities and subsequent cognitive impairment [45] if translational controls break down and allow for the increased DRD1 and 5 transcripts to produce more protein. Further, oxidative damage over time from PON2 deficiency may significantly contribute to cellular dysfunction and exacerbate the effects of abnormal dopamine receptor expression. Oxidative stress is a hallmark of neurodegenerative disease, with both Parkinson’s disease (PD) [19, 20] and Alzheimer’s disease (AD) [46]. Additional investigation of the life stages of PON2-def mice, as well as proteostasis controls, would be of interest, with a particular focus on PON2 deficiency in aged animals and potential neurodegenerative consequences.

In our study, PON2 expression was significantly increased upon exposure to 3 μM quinpirole, a DRD2/3 agonist, but not to 3 μM fenoldopam, a DRD1/5 agonist. Dopamine receptors are G-protein-coupled receptors and can be divided into two families based on their pharmacologic properties and effects on cAMP production: D_1_-like receptors (DRD1/5) and D_2_-like receptors (DRD2/3/4). While D_1_-like receptors couple to G stimulatory sites and increase cAMP production, D_2_-like receptors couple to G inhibitory sites and inhibit both cAMP production and activity of adenylate cyclase [47]. The different dopamine receptors (DRD1–5) also display overlapping and distinct functions, with many playing a role in locomotion, attention, and cognition [48]. Effects on locomotion may be of particular relevance, as we have observed that PON2-def mice display locomotor deficits when tested on the rotarod [9]. However, locomotion is a complex behavior and involves multiple pathways, and PON2 deficiency in this pathway alone may not be sufficient to affect rotarod performance. Behavioral assessment of DRD2 knockout mice has shown that observed locomotor deficits were largely driven by strain differences, and not the absence of DRD2 itself [49], supporting that perturbation in multiple genes is necessary for overt phenotypic effects. Additional work to elucidate the function of PON2 in the DRD2 signaling pathway would be of value to determine if disruption to this pathway is responsible for the locomotor deficits observed in PON2-def mice [9].

Previous studies utilizing immortalized human renal proximal tubule (RPT) and HEK293 cells have shown PON2 to be involved in DA receptor signaling, with exposure to 1 μM dopamine receptor agonist (DRD1/5 and DRD2/3) increasing PON2 expression [24, 25]. Although a higher concentration of 3 μM was required to elicit an increase in PON2 expression in our study, our results in primary mouse neurons show that, like kidney cells, PON2 expression was increased upon exposure to DRD2/3 agonist quinpirole. However, unlike kidney cells, no expression increase was observed upon exposure to DRD1/5 agonist fenoldopam. These data suggest there could be cell- and organ-specific differences in the role of PON2 in signaling. Conversely, these data may speak to important differences between immortalized versus primary cells, and/or species differences between humans and mice. The mechanism by which cells are immortalized involves disruption of normal cell proliferation, allowing cells to proliferate in perpetuity, which can alter their receptor function. As well, comparison of findings from different species can be difficult within the same cell type, much less across organ type. It would be of value to repeat our experimental conditions in primary mouse RPT cells to address organ-specific differences in PON2 DA signaling to eliminate many of these confounding factors.

No differences in DRD2 transcript or protein were observed in PON2-def mice compared to WT, supporting that PON2 is downstream of DRD2 and PON2 deficiency alone is not sufficient to alter DRD2 expression. This supports the existing literature examining PON2 levels in the kidney of DRD2 KO mice, where renal PON2 levels were 33% lower in DRD2 KO tissue compared to WT [24]. However, it is unclear whether PON2 plays a role in the presynaptic cell, postsynaptic cell, or both, as our methodology did not allow us to distinguish between DRD2 autoreceptor and postsynaptic receptor activation. Although the high concentration of quinpirole used in our study is expected to preferentially activate postsynaptic receptors, feasibly some autoreceptor activation is occurring, given the high affinity of DRD2 agonists for autoreceptors [50]. While the lack of significant PON2 modulation at a lower quinpirole concentration is encouraging that the observed increase is through postsynaptic binding, further exploration of the location of PON2 modulation would be of interest in future studies.

PON2 is primarily located to the mitochondrial membrane in the brain, although it has been additionally detected at the plasma membrane of neurons and astrocytes [6]. While the function at the plasma membrane is largely unknown, work in HEK 293 T cells has shown PON2 translocates to the plasma membrane under oxidative stress conditions to mitigate lipid peroxidation [51]. Although currently untested, PON2 may also translocate in neurons and astrocytes to interact with DRD2 at the synaptic membrane. Indeed, DRD2 and PON2 co-localize at the brush border of mouse renal proximal tubules [24], supporting extra-mitochondrial localization and direct DRD2 interaction in some cell types. Alternatively, PON2 may be upregulated in the mitochondria through DRD2 signaling cascades. In astrocytes, DRD2 has been found to modulate neuroinflammation by increased expression of αB-crystallin (CRYAB) [52]. DRD2 null mice are reported to have an increased inflammatory response and dopaminergic neuron sensitivity to the neurotoxic compound MPTP [52], potentially owing to a decrease in CRYAB expression. CRYAB, a member of the heat shock protein family, primarily locates to the mitochondria where it exerts anti-inflammatory and anti-apoptotic action [53]. While no direct interactions between PON2 and CRYAB have been reported in the literature, this may be a further avenue of study going forward to determine if astrocytic modulation of neuroinflammation through the DRD2-CRYAB pathway involves PON2. Of additional interest would be examination of PON2 expression in subcellular fractions upon DRD2 agonist exposure to determine if the upregulation of PON2 is at the plasma membrane or at the mitochondria level, a limitation that was not addressed in our current study.

## Conclusions

Evidence supports PON2 as a critical antioxidant enzyme in the brain, however, studies on its role in the CNS have been limited to date. Our study addresses key gaps in the literature regarding the effects of PON2 deficiency in the dopaminergic system of the CNS and demonstrates that PON2 deficiency significantly upregulates the transcript of multiple dopaminergic related genes in the striatum of mice. As well, our study identified potential organ-specific upregulation of PON2 through selective dopamine receptor signaling. Given the findings of this study, further investigation into PON2 in the dopaminergic system would be of interest to examine downstream consequences of these observed transcript changes and probe further into the role of PON2 in dopamine receptor signaling in the brain.

## Supplementary Information

**Additional file 1.** NormFinder results.

**Additional file 2.** Monoamine oxidase B (MAOB) results and western blot raw data.

## Data Availability

All data generated and analyzed during the current study are available from the corresponding author on reasonable request.

## Abbreviations

CNS: Central nervous system
PON2: Paraoxonase 2
ROS: Reactive oxygen species
WT: Wildtype
PON2-def: Paraoxonase 2 deficient
DRD(1/2/3/5): Dopamine receptor (1/2/3/5)
HDL: High density lipoproteins
DA: Dopamine
PD: Parkinson’s disease
AD: Alzheimer’s disease
6-OHDA: 6-Hydroxydopamine
MPTP: 1-Methyl-4-phenyl-1,2,3,6-tetrahydropyridine
SNc: Substantia nigra pars compacta
PND: Postnatal day
CGN: Cerebellar granule neuron
TH: Tyrosine hydroxylase
VMAT2: Vesicular monoamine transporter 2
DAT: Dopamine transporter
HO-1/2: Heme-oxygenase 1/2
NOX2: NADPH oxidase 2
MAOA/B: Monoamine oxidase A/B
miRNA: Micro RNA
RPT: Renal proximal tubule
CRYAB: αB-crystallin
