# Supplementary material for: Examining the role of paraoxonase 2 in the dopaminergic system of the mouse brain
Source: BMC Neurosci. 2022 Sep 2;23:52. doi: 10.1186/s12868-022-00738-4 (PMC9438175; doi:10.1186/s12868-022-00738-4)

Additional File 2

MAOB Expression

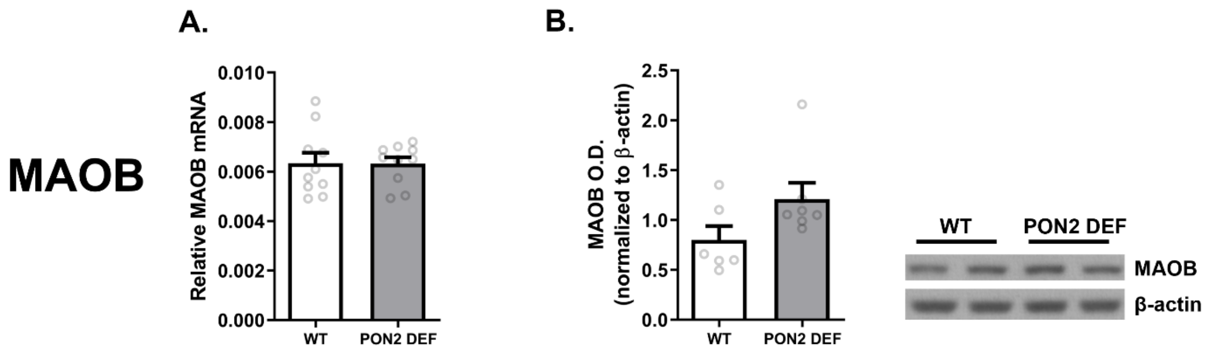

A. Quantification of monoamine oxidase B (MAOB) mRNA normalized to GAPDH, mean (± SEM), n = 8 – 10 per group. **B.** Quantification of monoamine oxidase B (MAOB) protein normalized to β-actin, mean (± SEM), n = 9 - 10 per group.

Western blot raw data

Note: All band examples from manuscript figures are denoted with a red box

Paraoxonase 2 (PON2)

PON2 (42 kDa)

(1 – 3 WT F, 4 – 6 PON2 DEF F, 7 – 9 WT M, 10 – 12 PON2 DEF M)

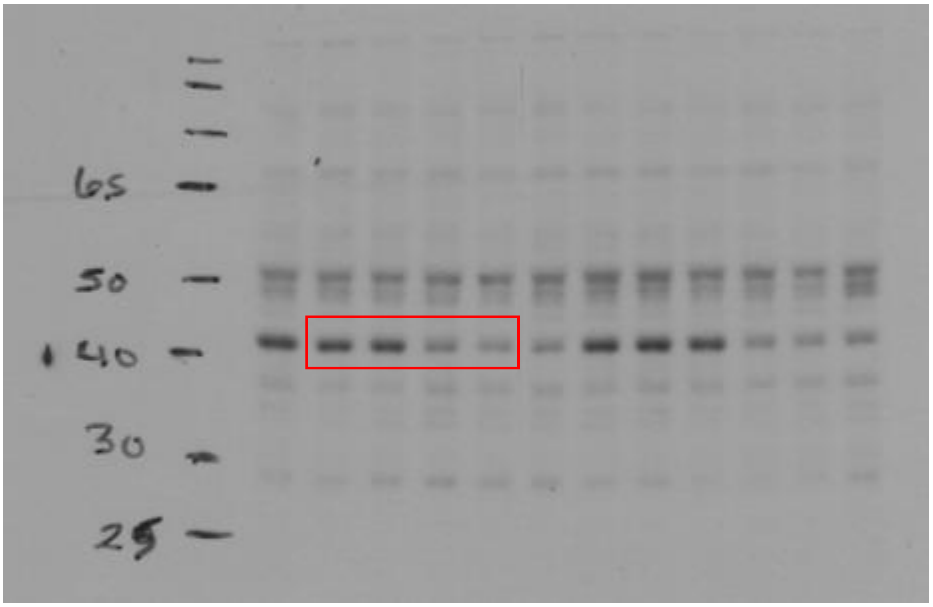

β-actin (42 kDa)

(1 – 3 WT F, 4 – 6 PON2 DEF F, 7 – 9 WT M, 10 – 12 PON2 DEF M)

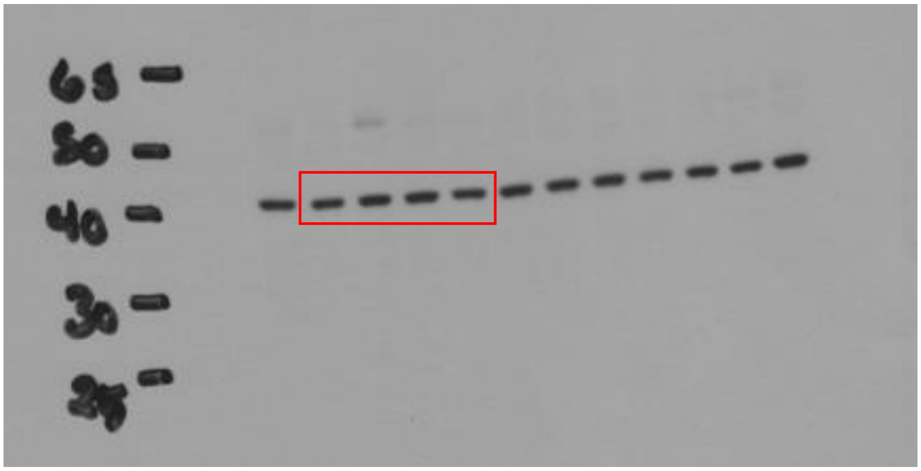

Dopamine receptor 1 (DRD1)

DRD1 (~49 kDa)

(1 – 3 WT F, 4 – 6 WT M, 7 – 9 PON2 DEF F, 10 – 12 PON2 DEF M)

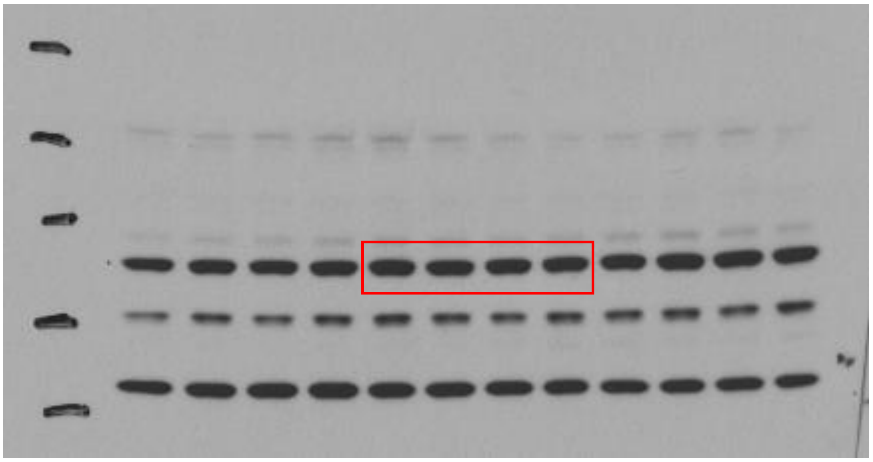

β-actin (42 kDa)

(1 – 3 WT F, 4 – 6 WT M, 7 – 9 PON2 DEF F, 10 – 12 PON2 DEF M)

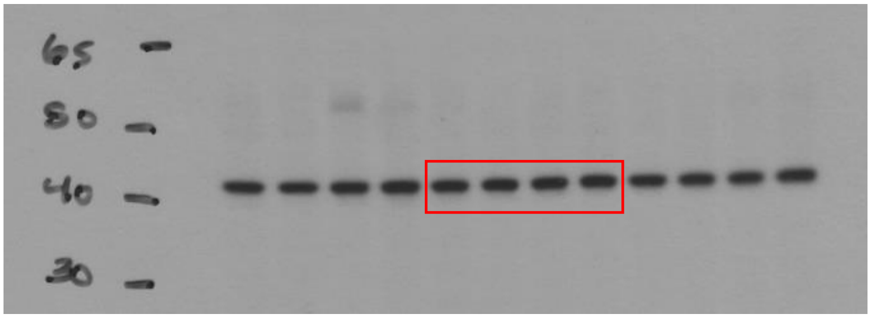

Dopamine receptor 2 (DRD2)

DRD2 (~50 kDa)

(1 – 5 WT F, 6 – 10 PON2 DEF F)

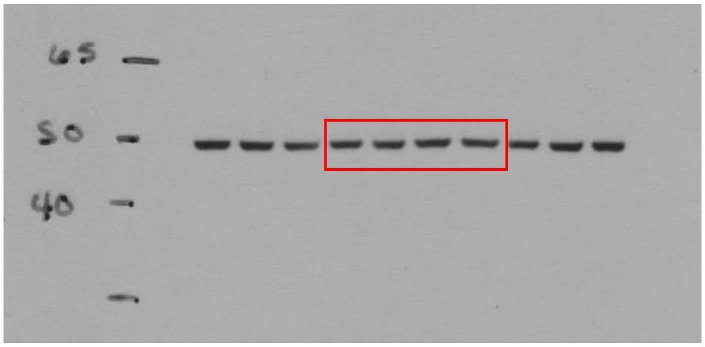

(1 – 5 WT M, 6 – 10 PON2 DEF M)

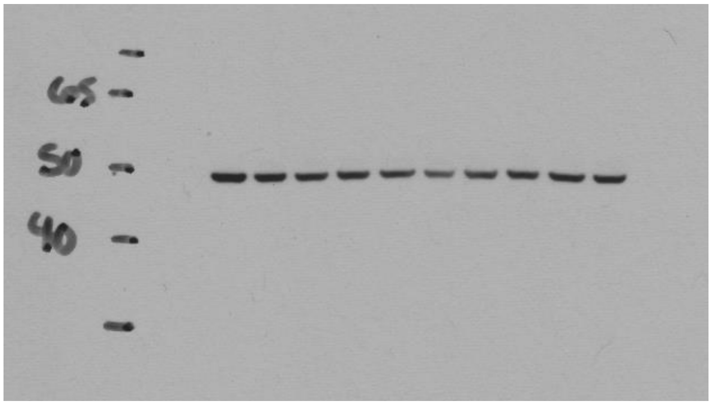

β-actin (42 kDa)

(1 – 5 WT F, 6 – 10 PON2 DEF F)

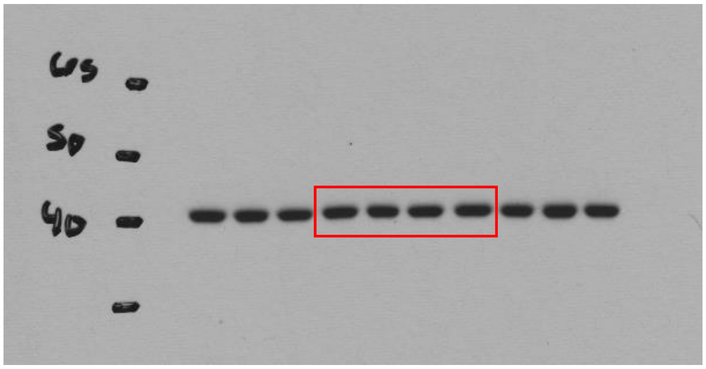

(1 – 5 WT M, 6 – 10 PON2 DEF M)

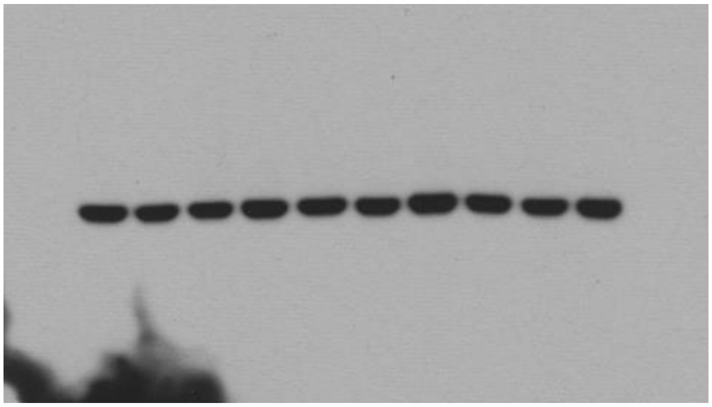

Dopamine receptor 5 (DRD5)

DRD5 (~50 kDa)

(1 – 5 WT F, 6 – 10 PON2 DEF F)

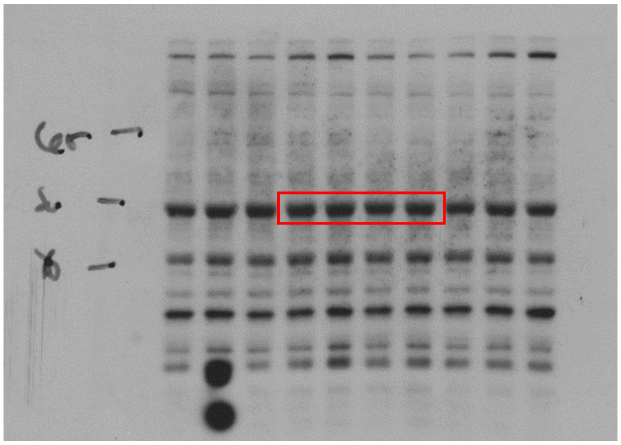

(1 – 5 WT M, 6 – 10 PON2 DEF M)

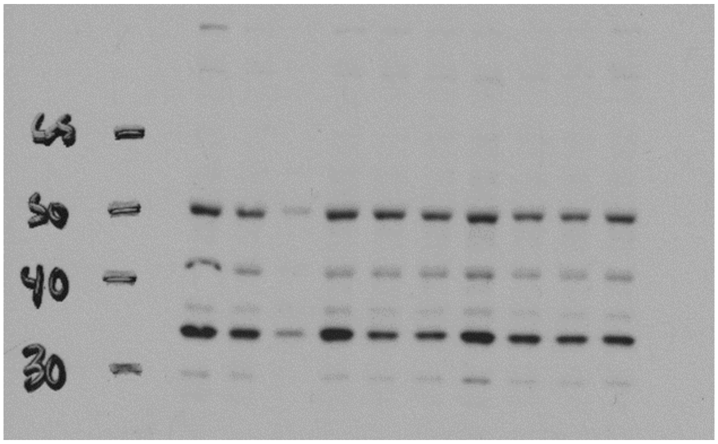

β-actin (42 kDa)

(1 – 5 WT F, 6 – 10 PON2 DEF F)

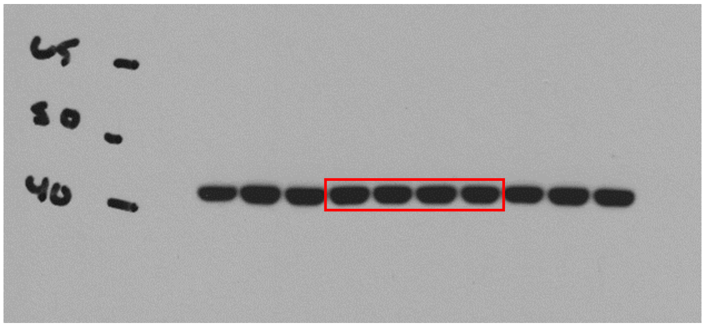

(1 – 5 WT M, 6 – 10 PON2 DEF M)

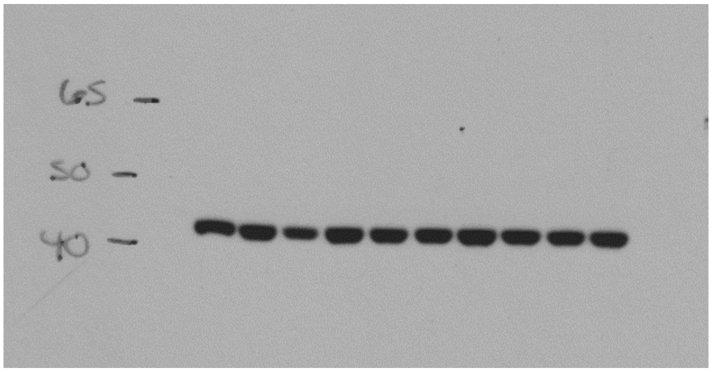

Tyrosine hydroxylase (TH)

TH (60 kDa)

(2 – 6 WT F, 1 & 7 – 10 PON2 DEF F)

*** Sample #1 PON2 DEF due to loading order error**

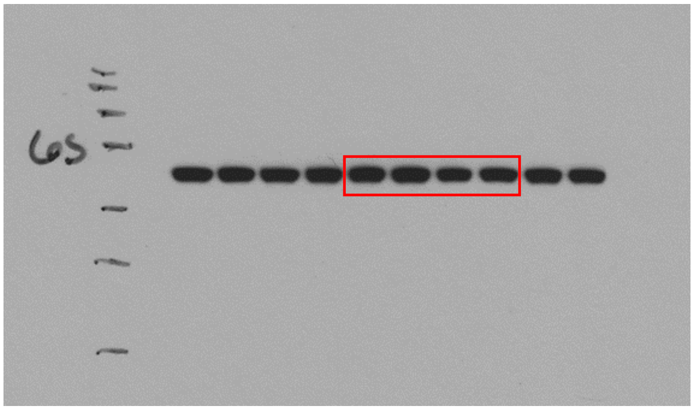

(1 – 5 WT M, 6 – 10 PON2 DEF M)

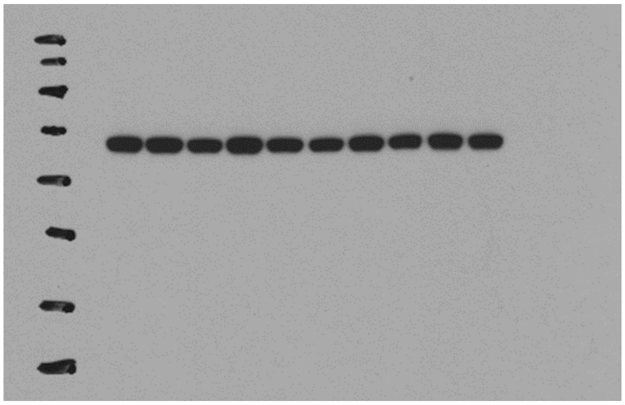

β-actin (42 kDa)

(1 – 5 WT F, 6 – 10 PON2 DEF F)

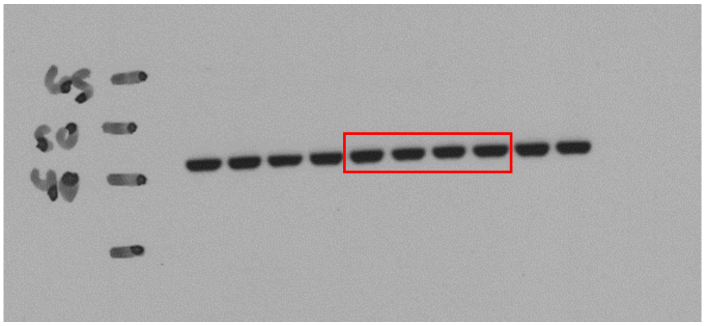

(1 – 5 WT M, 6 – 10 PON2 DEF M)

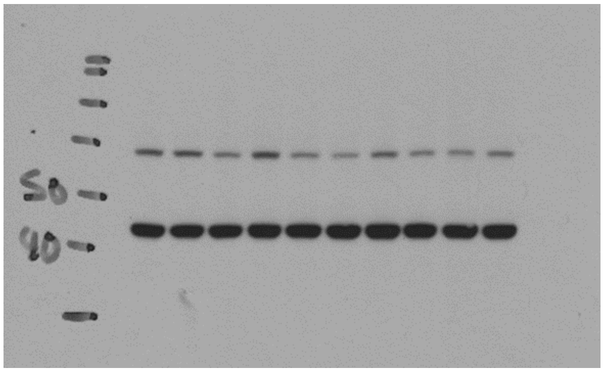

<u>**Vesicular monoamine transporter 2 (VMAT2)**</u>

VMAT2 (~50 kDa)

(1 – 5 WT F, 6 – 10 PON2 DEF F)

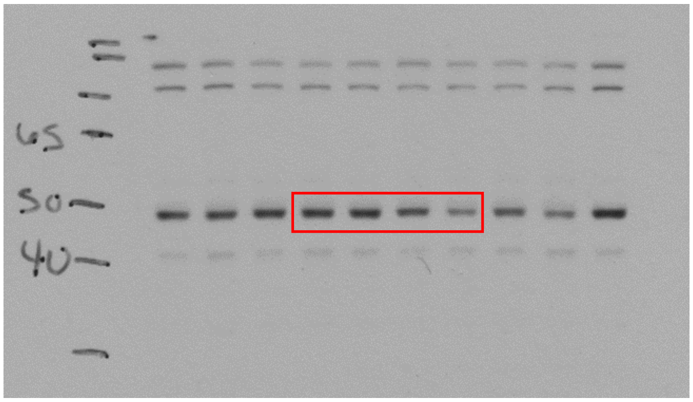

(1 – 5 WT M, 6 – 10 PON2 DEF M)

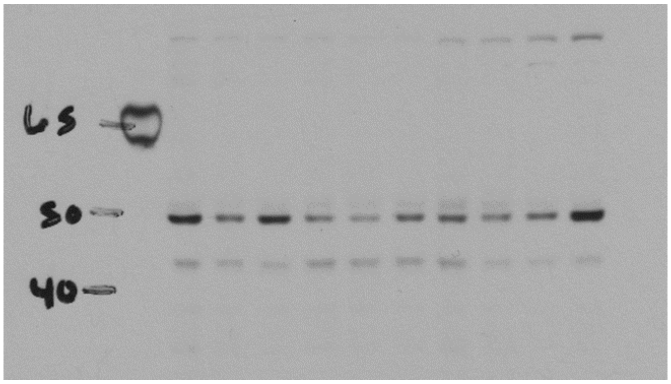

β-actin (42 kDa)

(1 – 5 WT F, 6 – 10 PON2 DEF F)

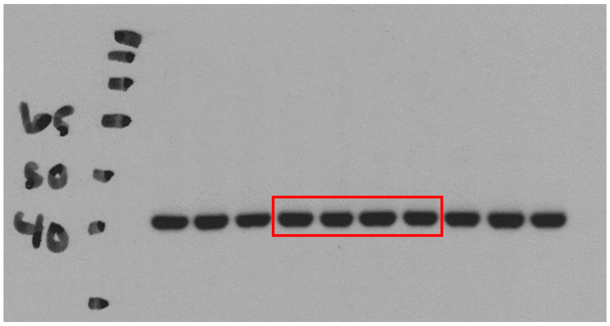

(1 – 5 WT M, 6 – 10 PON2 DEF M)

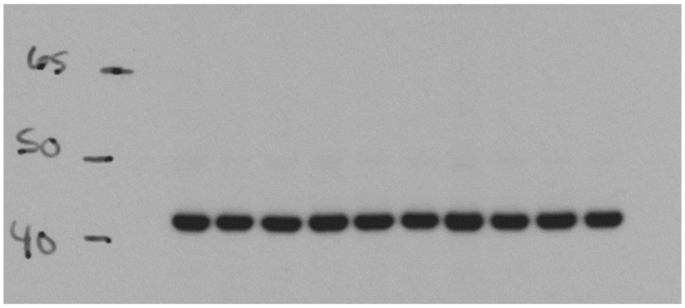

Dopamine transporter (DAT)

DAT (~70 kDa)

(1 – 3 WT F, 4 – 7 WT M, 8 – 10 PON2 DEF F, 11 – 14 PON2 DEF M)

~70 kDa

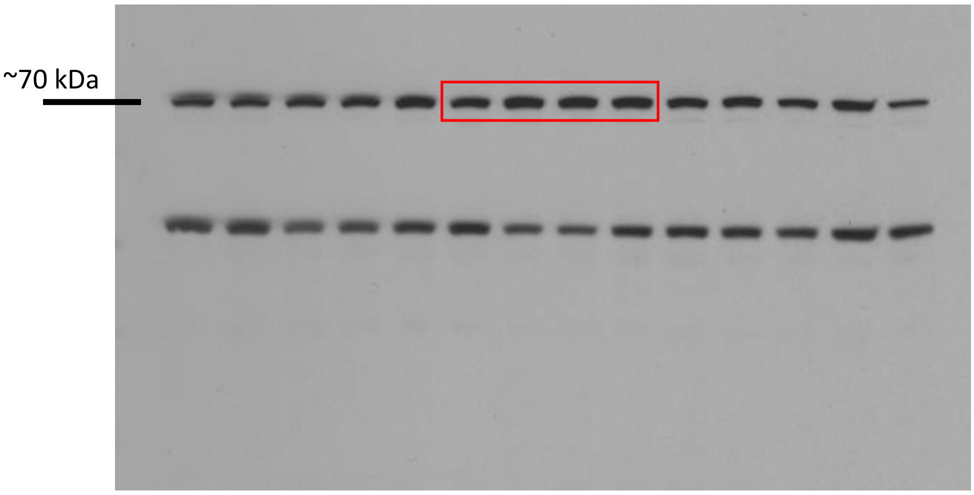

β-actin (42 kDa)

(1 – 3 WT F, 4 – 7 WT M, 8 – 10 PON2 DEF F, 11 – 14 PON2 DEF M)

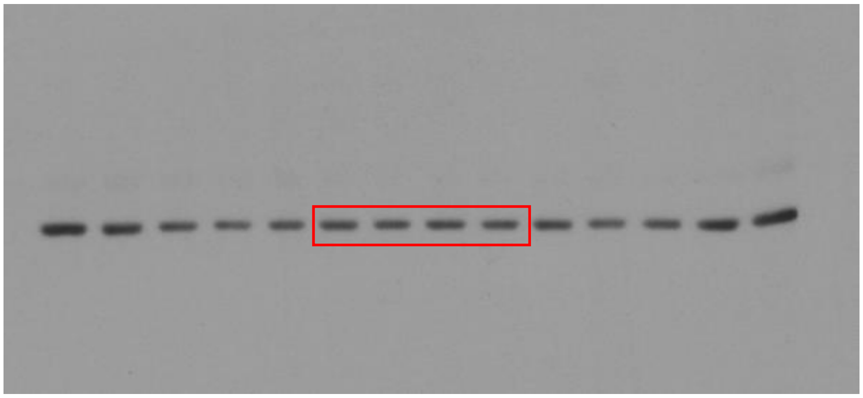

Monoamine oxidase B (MAOB)

Note: Bands in Figure from Additional File 2 are horizontally flipped to demonstrate WT samples on left side

MAOB (~58 kDa)

(1 – 3 WT F, 4 – 6 PON2 DEF F, 7 – 9 WT M, 10 – 13 PON2 DEF M)

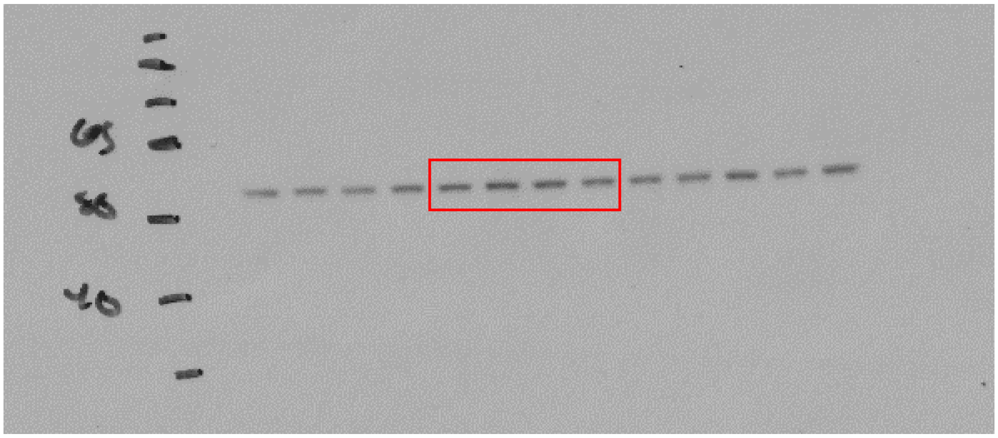

β-actin (42 kDa)

(1 – 3 WT F, 4 – 6 PON2 DEF F, 7 – 9 WT M, 10 – 13 PON2 DEF M)

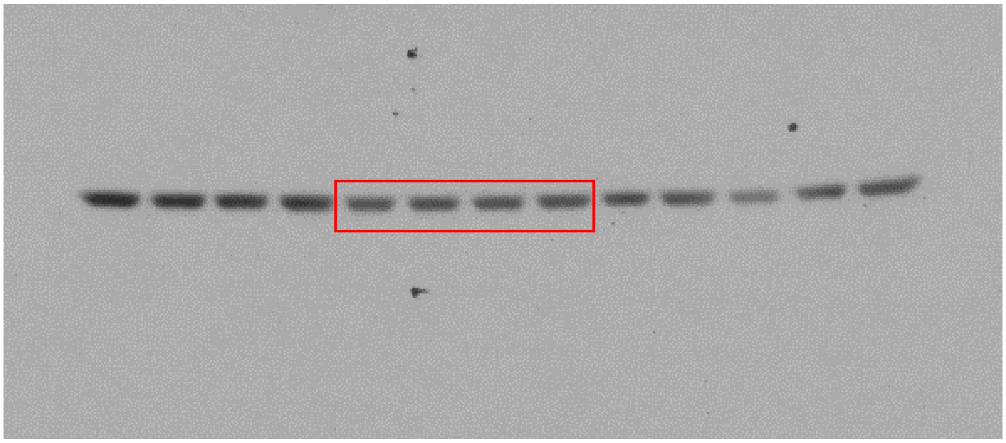

Heme oxygenase 1 (HO-1)

Note: Non-adjacent bands used in manuscript figure 4B to better reflect overall expression profile per reviewer comment

HO-1 (~33 kDa)

(1 – 5 WT F, 6 – 10 PON2 DEF F)

(1 – 5 WT M, 6 – 10 PON2 DEF M)

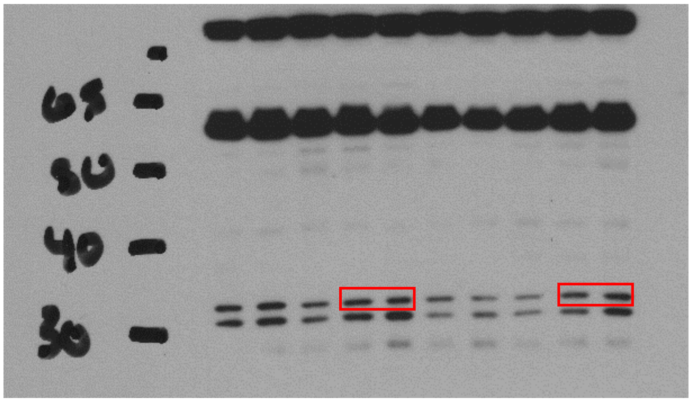

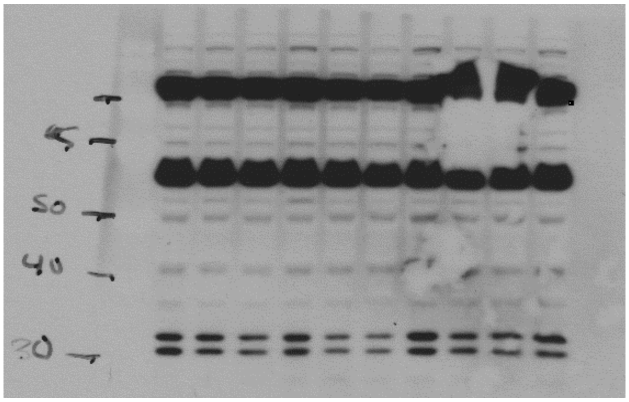

Note: Samples #7 and 8 on right blot were dropped from analysis due to bubble

β-actin (42 kDa)

(1 – 5 WT F, 6 – 10 PON2 DEF F)

(1 – 5 WT M, 6 – 10 PON2 DEF M)

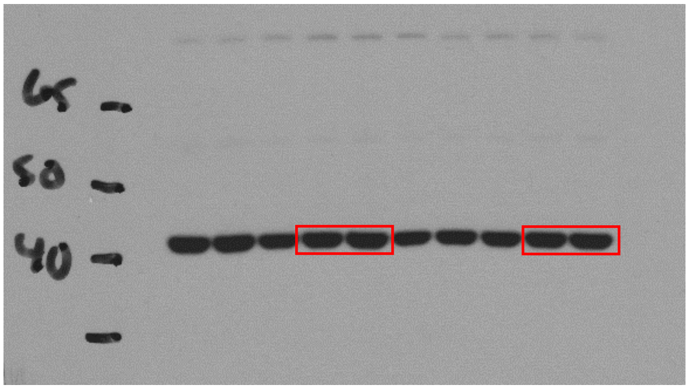

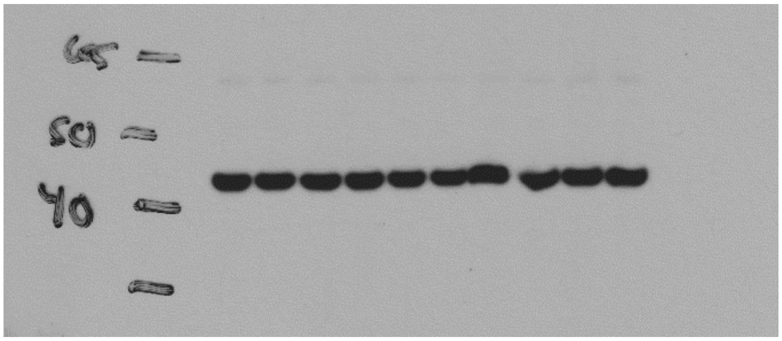

Heme oxygenase 2 (HO-2)

HO-2 (~35 kDa)

(1 – 5 WT F, 6 – 10 PON2 DEF F)

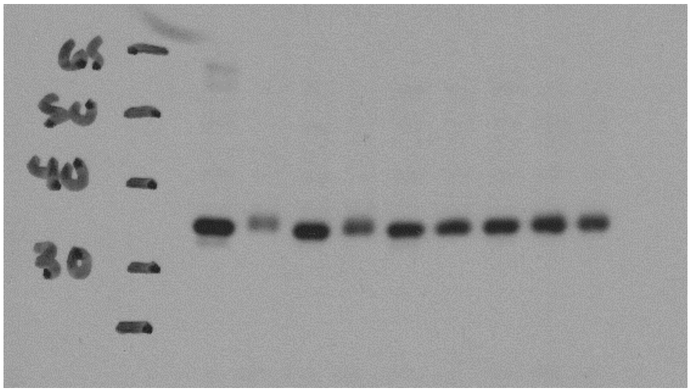

(1 – 5 WT M, 6 – 10 PON2 DEF M)

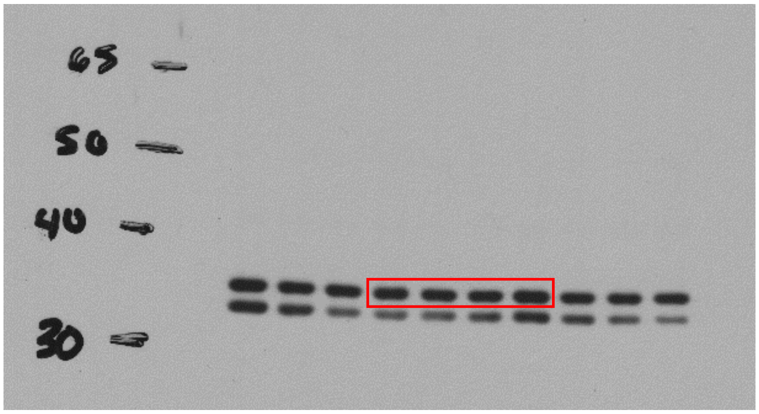

β-actin (42 kDa)

(1 – 5 WT F, 6 – 10 PON2 DEF F)

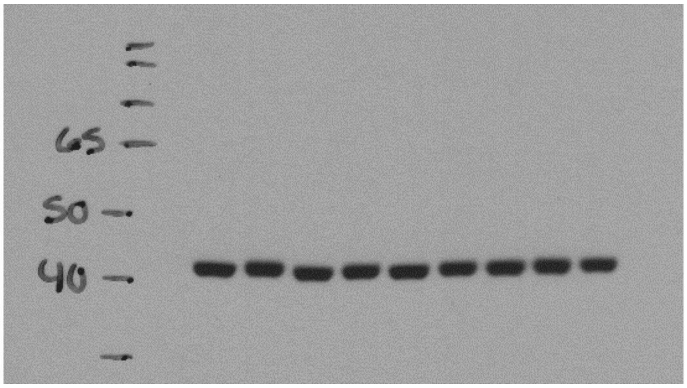

(1 – 5 WT M, 6 – 10 PON2 DEF M)

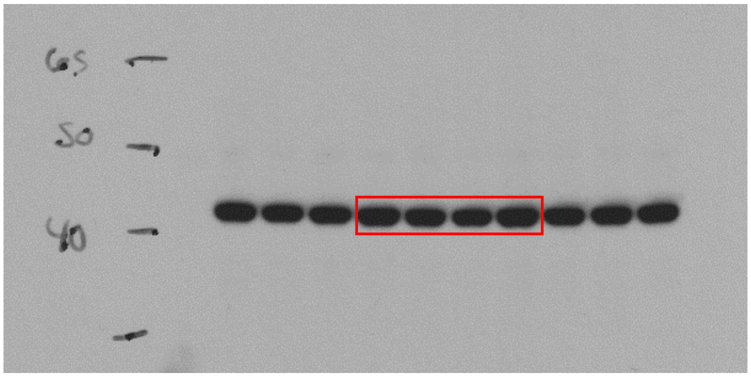

NADPH oxidase 2 (NOX2)

NOX2 (~60 kDa)

(1 – 5 WT F, 6 – 10 PON2 DEF F)

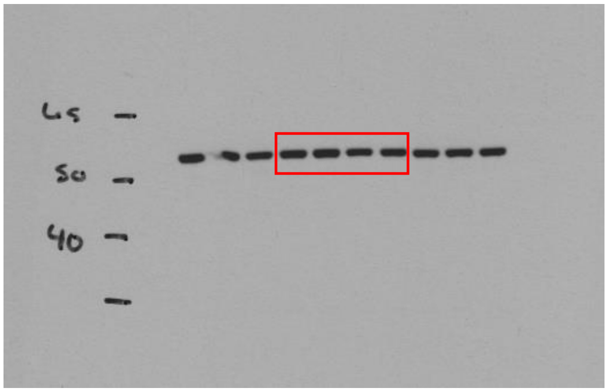

(1 – 5 WT M, 6 – 10 PON2 DEF M)

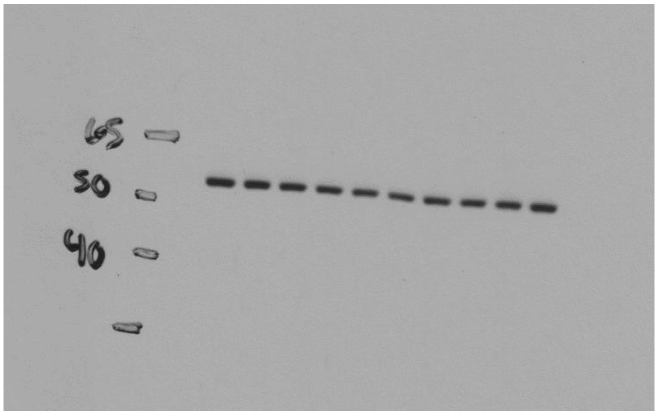

β-actin (42 kDa)

(1 – 5 WT F, 6 – 10 PON2 DEF F)

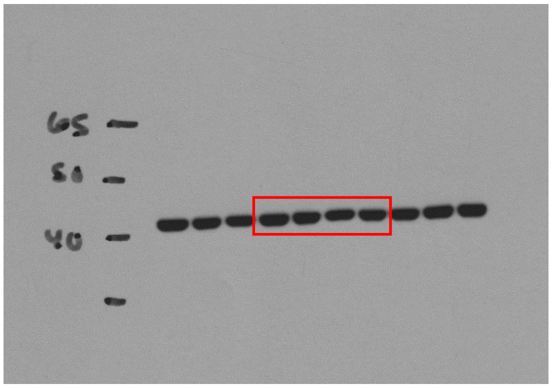

(1 – 5 WT M, 6 – 10 PON2 DEF M)

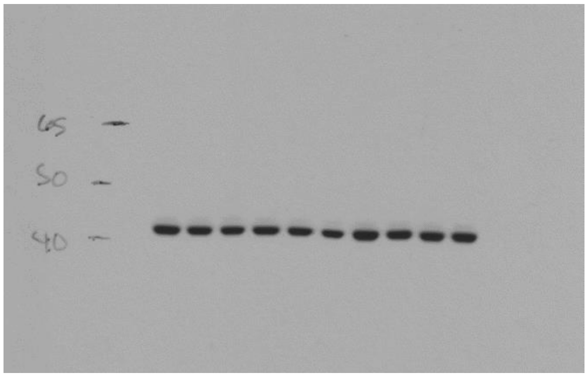

Paraoxonase 2 expression after fenoldopam exposure

Note: Bands in Figure 5 of manuscript were elongated and enlarged to fit figure size

PON2 (42 kDa)

(1 , 3, 5, 7 vehicle control) (2, 4, 6, 8 3 µM fenoldopam 24 hour exposure)

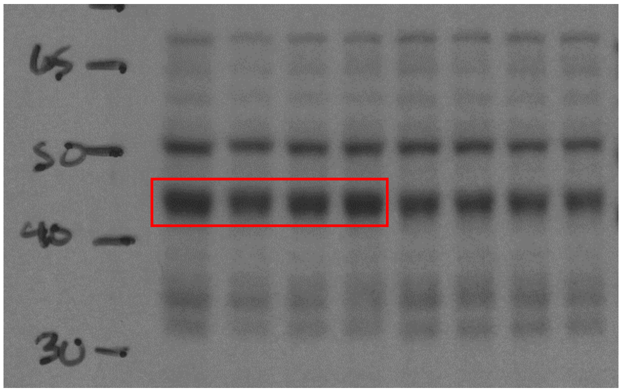

β-actin (42 kDa)

(1 , 3, 5, 7 vehicle control) (2, 4, 6, 8 3 µM fenoldopam 24 hour exposure)

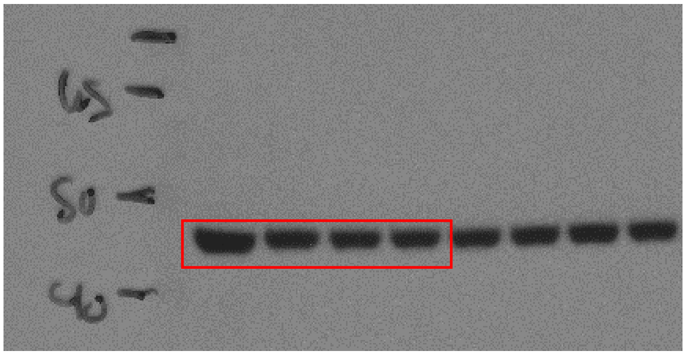

Paraoxonase 2 expression after quinpirole exposure

PON2 (42 kDa)

Blot 1 (1,5 VC, 2,6 L-741,626, 3,7 L-741,626 + quinpirole, 4,8 3 µM quinpirole)

Blot 2 (1,5 VC, 2,6 L-741,626, 3,7 L-741,626 + quinpirole, 4,8 3 µM quinpirole)

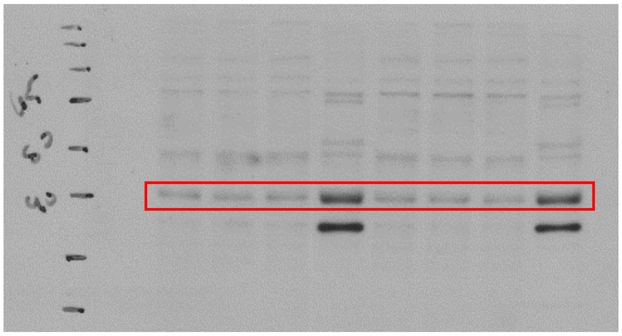

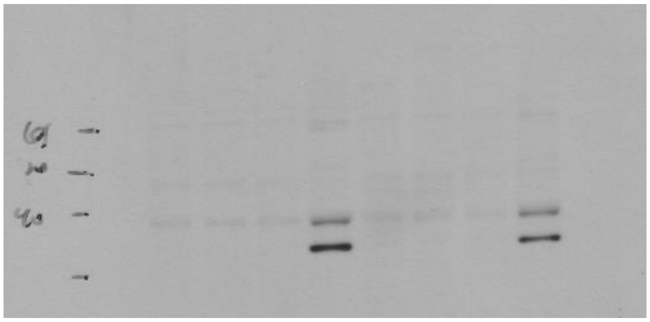

β-actin (42 kDa)

Blot 1 (1,5 VC, 2,6 L-741,626, 3,7 L-741,626 + quinpirole, 4,8 3 µM quinpirole)

Blot 2 (1,5 VC, 2,6 L-741,626, 3,7 L-741,626 + quinpirole, 4,8 3 µM quinpirole)

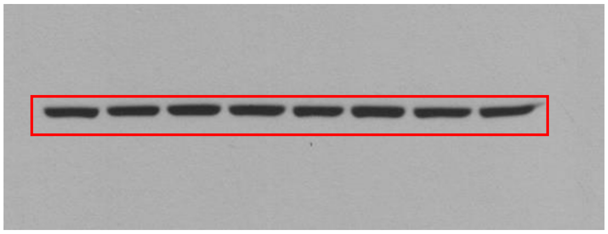

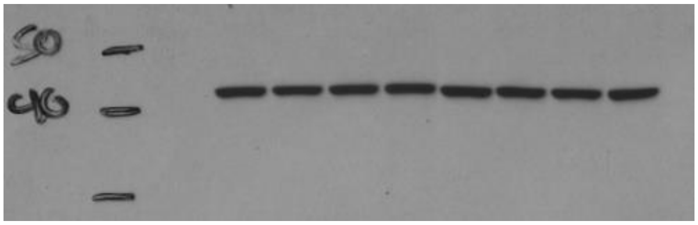

Supplement: Supplementary file 2 — Additional file 2. Monoamine oxidase B (MAOB) results and western blot raw data. [file 12868_2022_738_MOESM2_ESM.pdf]
